# On the Autopsy of Epidemic Cholera

**Published:** 1833-07

**Authors:** Alexander Turnbull Christie

**Affiliations:** the Honourable East-India Company's Service; Member of the Royal Asiatic Society of Great Britain and Ireland, of the Wernerian and Royal Medical Societies of Edinburgh, &c.


THE EPIDEMIC CHOLERA.

*On the Autopsy of Epidemic Cholera.* By ALEXANDER TURNBULL CHRISTIE, M.D., of the Honourable East-India Company's Service; Member of the Royal Asiatic Society of Great Britain and Ireland, of the Wernerian and Royal Medical Societies of Edinburgh, &c.

IT is a prevalent opinion that no post-mortem appearances have been observed in cholera, which could enable us to infer what were the diseased actions which had existed during life, and had constituted the disease. I apprehend, however, that, in many dissections, the examination of the various important parts of the body has been very partial,* and that the nature of some of the morbid appearances has been misunderstood.

It is well known that different visitations of cholera have been characterized by the unusual prevalence, or predominance, of certain symptoms; that Europeans, from their plethoric and irritable habits, are more liable to that type of the disorder which is distinguished by pain and burning at the stomach, violent spasms, and which is sometimes ushered in by increased action; and, lastly, that the abstemious Hindoo is particularly subject to the low and most dangerous form of the malady. It is quite evident, therefore, that if any one derives his notions of the autopsy of the disease from his own observations alone, from one visitation, or from one class of patients, they are most likely to be imperfect or erroneous; and that the only method of arriving at correct views of its pathology is to examine carefully the morbid changes that have been produced by it, in all its forms, in every description of patient, and at different times and places. But it is unfortunate that medical men have often been prevented from ascertaining the morbid changes produced by the disease in the bodies of native patients, by the religious prejudices of the Hindoos and Mahometans against dissection.

I was enabled to obtain an accurate knowledge of the post-mortem appearances in native subjects, when civil-surgeon at

* A medical friend once told me that he had opened the bodies of several subjects who had died of cholera, but that he had found no morbid appearances which could account for the disease. Upon asking him whether he had examined the mucous membranes carefully, he said that in no case had he thought of looking at any one of them. This plainly shews that the autopsy of cholera has not received that degree of attention which it deserves. We sometimes find it stated, in the relation of cases, that no morbid appearances could be detected on dissection sufficient to account for the death of the patient; but if the mucous membranes, and perhaps also other important organs, were not examined, of what use is such an observation?

Darwar, where my favourable situation, and the assistance I received for this purpose from the civil authorities, afforded me an opportunity of making numerous dissections.* I have consulted Mr. Annesley's work with much advantage, in reference to the morbid changes observed in the bodies of Europeans; have examined all the pathological details contained in the Madras and Bengal Reports, and in other works on Cholera, and can therefore confidently affirm that the following observations may be depended upon as being perfectly accurate.

Many of the morbid changes which have been described by different authors are by no means uniform, and, excepting a few connected with the mucous system, and with the state of the blood, none can be said to be invariable.

*Encephalon.* Great congestion in the veins and sinuses of the brain, and of its membranes, has been generally observed in post-mortem examinations in this disease. Cases have been mentioned in which the tunica arachnoidea has been found thickened, and adhering to the adjacent membranes, and in which the substance of the brain has been soft and pulpy;† and a gelatinous or serous effusion has been often observed in the ventricles and between the membranes. Traces of inflammation are said to have been seen in the brain, in the tuber annulare, or in the spinal marrow; but they could not be detected in any of the cases that have fallen under my own observation; they are not mentioned by Annesley, or in the greater number of Reports sent by medical officers to the Medical Boards, and must therefore be considered as not belonging at all to the disease, or to be of very rare occurrence. Of all these morbid appearances, venous congestion is the most frequent; but it also is sometimes wanting, many cases having been observed in which the contents of the cranium have been in every respect perfectly healthy.

*Thorax.* No changes of any importance have been observed in any of the serous membranes of the thorax. A good deal of venous congestion has been generally found in the lungs; and their substance has often presented a bruised, hepatised, or fleshy appearance. They have been described by

* "Dissections have been chiefly made on the bodies of European soldiers, a class of men acknowledged to be peculiarly liable in this climate to visceral disease of all kinds. Under these circumstances, dissection reports should be received with care, in reference to the general states of morbid bodies, and with the most attentive consideration of the precise import of the terms employed." —*Madras Report.*

† This appearance has not fallen under my own observation, and is probably of rare occurrence.

some authors to have been shrunk and drawn back, as it were, to the spine; but this could not have taken place during life, but at the time only when the thorax was opened; for it is impossible to conceive that the lungs could have exerted a power sufficient to overcome the whole weight of the atmosphere, and to leave the cavity of the pleura empty; or if, on the other hand, this cavity had by any means become distended with air, suffocation must have instantly been the result. I will defer saying any thing of the state of the mucous membrane of the lungs, until I come to treat of the morbid condition of the mucous membranes generally.

The large vessels leading to the heart, and the right auricle and ventricle, have almost always been found gorged with black blood, which has also, in some instances, extended to the left auricle. Here will be the proper place for describing those extraordinary changes in the blood which form one of the most important and prominent features of the disease, and which are perhaps always present in the latter stages. The following are the results of my observations on it, as drawn by venesection, by leeches, or as seen after death, in a great variety of cases. Sometimes it has been perfectly black, of the consistence of liquid honey, or forming a uniform coagulum after a few minutes' exposure to the air; and these appearances it has retained for twenty-four hours, without separating into serum and crassamentum. In some cases it has been darker than usual, has not become fluid, but has coagulated, and separated a good deal of serum. I have observed it of the usual dark colour, with red streaks; and these streaks appeared to increase in some instances as the bleeding was continued; and lastly, I have seen it quite natural, except perhaps being a little darker than usual when first drawn.* The veins of some of the internal viscera were always found distended with dark blood, the vessels at the surface being nearly empty.

*Abdomen.* On opening the abdomen, a peculiar offensive odour has been sometimes observed to proceed from it. . Mr. Annesley says, the omentum was sometimes corrugated, or thrown to one side. The only diseased appearance which has been found in the peritoneal surface of the stomach and intestines is a blue or purplish colour, from venous congestion. A vermilion tint also sometimes occurs, and has been attributed to inflammation, but improperly, for it is evidently owing to the distention of the very minute ramifications of the

* It is worthy of remark, that black-coloured blood is not peculiar to cholera. I have seen blood taken from rheumatic and dysenteric patients, in India, continue black for many hours after it was drawn.

veins, which, with a little attention, can be traced to the large venous trunks. There is, I believe, invariably great venous congestion in many of the abdominal viscera, but some of them are generally more affected than others: for instance, great congestion has been observed on the surface of the intestines, and at the same time none on that of the stomach; portions of the intestines are often quite free from congestion, in other instances completely discoloured by it. I have never seen any venous congestion in the urinary bladder, which is almost always contracted, and not otherwise altered in its external appearance. The stomach is very frequently distended, sometimes it is contracted. It generally happens that some portions of the intestines are thin and greatly distended with the cholera secretion, or with flatus, while other portions are very much contracted, thickened, and possessing a doughy feel. The liver is generally loaded with dark-coloured blood; but many instances occur in which it does not contain more blood than usual, and presents a perfectly healthy appearance. The following observations on the state of the bile are extracted from Mr. Annesley's work.

"The gall-bladder was always distended by thick viscid bile, which was generally of a dark-green or black colour, in subjects who died before the appearance of bile in the excretions; and, although the hepatic duct was large and permeable, the mouth of the common duct was generally constricted, and seldom permitted the bile to flow into the duodenum without considerable pressure made upon the gall-bladder. In those cases which terminated fatally after an illness of long duration, and in which some reaction of the vital energies and a flow of bile into the intestines had taken place, the gall-bladder was generally empty, or contained but a small quantity of bile; and the common duct, although not always free from some degree of constriction, was generally more permeable than in the former class of cases. In a few instances the gall-bladder was quite empty, relaxed, and flabby. In almost all the cases wherein bile was observed in the excretions, and the gall-bladder was found empty on dissection, and consequently when it could be legitimately inferred that this secretion had passed into the intestines during the life of the patient, I remarked that the viscid matter usually found lining the mucous surface of the small intestines, in the former description of cases, was detached to a greater or less extent, and was either floating in the fluid contents of the large intestines, or entirely removed along with the matters which had been ejected from them."

The spleen was generally gorged with black blood, and its texture was often soft and loose. In one remarkable case,* however, which came under my own observation, it was soft, flabby, and *did not contain a drop of blood.*

No morbid appearances have been noticed in the kidneys or pancreas.

I will now proceed to describe the morbid appearances which have been observed in the mucous membranes, which, from their constancy and great extent, must be considered as the most important connected with this disease. These membranes had an unnatural whiteness; were frequently soft and pulpy; and in general, especially in the stomach and intestines, could be easily detached by scraping from the subjacent coat. In some situations, a whitish, opaque, viscid substance was found adhering to their surface, and was often so abundant in certain parts of the intestines as completely to fill them. The different cavities lined by mucous membranes contained more or less of a transparent or turbid serous fluid; the viscid matter already mentioned being often found intimately mixed with it, or floating in it in the form of flakes, giving rise to the different kinds of the cholera secretion already alluded to in the first chapter. These appearances were sometimes more or less partial, but some of them were generally found throughout the whole extent of the alimentary canal; they extended in many cases to the mucous membrane of the bladder and ureters; and I have observed them, in two or three instances, in the pulmonary mucous membrane.

The venous congestion already mentioned often extended to some parts of the internal surface of the stomach and intestines, which then exhibited patches or spots of blue, purple, or vermilion, according to the intensity and extent of the congestion.

It will be necessary to describe the morbid appearances observed in the different parts of the mucous membranes a little more in detail. That of the stomach was often perfectly white throughout its whole extent; a vermilion blush has been often observed at the pyloric extremity, and has been referred to inflammation, but which has been more probably owing to venous congestion. Purple patches on the internal surface of the stomach were not uncommon, and when the congestion had proceeded to a great extent, it gave rise to an appearance of ecchymosis. Calomel has often been found adhering to the inner coat of the stomach, and surrounded with a ring of a reddish colour. The contents of the stomach varied very much in their appearance, from the mix-

* Yella's case, in the Appendix.

ture of the medicines given to the patient with the cholera secretion; and food has sometimes been found along with them when the case has been rapid, and the vomiting trifling.

The mucous coat of the small intestines, like that of the stomach, was always found unusually white, excepting when discoloured by congestion; it was thickened where the gut was contracted, thinner than usual where the gut was distended, and was always soft, sometimes pulpy, and easily detached. In many works on cholera, a considerable degree of congestion of a blue, purple, or vermilion colour is mentioned as of very common occurrence, on the inner surface of the small intestines; but in my own practice it has been more frequently absent than present. There was often a very large quantity of the cholera secretion in this part of the alimentary canal, and a great abundance of the white viscid matter adhering to the villous coat. " When the disease was of longer continuance (says Mr. Annesley), and more particularly when some reaction of the powers of the system had taken place, this viscid appearance was detached to a greater or less extent, and was floating in the fluid contents of the small and large intestines; and the mucous coat then seemed more vascular, and the arterial capillaries more injected, than in the former class of cases.*"

Nearly the same observations apply to the large as to the small intestines; they were sometimes contracted, sometimes much distended, and their mucous membrane was either white, or more or less deeply coloured by venous congestion.

Worms, especially the lumbricus teres, have been found in the intestines after death, in a large proportion of cases, and have been often passed by vomiting and stool; and I think it highly probable that the peculiar condition of the gastro-enteric mucous membrane, which is favourable to the existence of these animals, may act as a predisposing cause of the disease.

The urinary bladder was always contracted, never containing any urine, but frequently a considerable quantity of the cholera secretion. Its mucous membrane, and also that of the ureters, was invariably white, and sometimes covered with a white viscid substance, similar to that in the alimentary canal.†

* Cases which had proved suddenly fatal.

† It has been observed by Bichat, that the various epidemic catarrhs described by authors have been generally characterized by the disorder being confined to the gastro-pulmonary membrane, the genito-urinary remaining unaffected. Cholera, however, forms an exception to this general rule; for in it the catarrhal affection frequently extends to every mucous membrane of the body.

Venous congestion is sometimes met with, but is not so common in the urinary bladder as in the different parts of the digestive tube.

For some time, believing the stomach and intestines to be the only seats of the disease, I unfortunately overlooked the condition of the pulmonary mucous membrane, and, since my attention has been directed towards the latter, I have had it in my power only two or three times to ascertain the state of that membrane by dissection. The first of the cases alluded to was that of an old man, a convict, who died of cholera after an illness of about ten hours. The symptoms were frequent watery purging, collapsed features, gradual diminution in the size of the pulse, coldness of the extremities, and difficulty of breathing. Having been attacked with the disease during the night, and not having reported his illness till the morning, the remedies came too late, and the disease proved fatal. The usual morbid appearances were found in the gastro-enteric mucous membrane, with little venous congestion in the abdomen, and no inflammation. The trachea was lined with a thickish mucus, and the minute branches of the bronchi were completely filled with a white froth. The second case being in many respects extremely interesting, and the dissection having been made with great care, in the presence of a medical friend, I will relate it in detail.

15th June, 1826. Anomah, male convict, aged twenty, was brought into hospital about half-past five P.M., from Moogud, a village five miles from Darwar.

Six P.M. It is reported that he vomited and was purged two or three times early this morning; but he himself positively asserts that he neither vomited nor was purged. When interrogated, he complains of nothing but slight pains in his limbs. His intellect is perfectly clear, and, although he is weak, he has a perfect command over all his voluntary muscles. Features considerably collapsed; pulse not perceptible at the wrist or temples; no perspiration on any part of the body; hands and feet cold; tongue coated, whitish, and moist.—Sumat statim Submur. Hydrarg. ℈i., et superbibat Tincturæ Cardamomi ℥i, in paululo aquæ tepidæ. Admoveantur sinapismi pedibus et cruribus, emplastrum epispasticum forte abdomini et arena calida brachiis.

Seven P.M. Has taken two doses of the calomel and tincture of cardamoms. Says he feels a little better. Skin colder since last report; features much collapsed. Complains of slight pains in his knees, and of pain from the cataplasms. Answers all questions most distinctly.—Sumat Submur. Hydrargyr. gr. v., necnon Tinct. Cardamom ʒiij., in paululo aquæ tepidæ quaque semihora.

Nine P.M. Has taken four doses of the calomel and tincture of cardamoms. Is restless, and complains of thirst. Pain in the umbilical region on pressure; pain in his knees continues; skin cold, with a slight cold perspiration; no pulse at the wrist or temples.—Continuantur remedia.

Ten P.M. Has coughed up a quantity of white froth; in other respects the same.

He died about midnight, and the body was examined at six o'clock on the following morning.

*Abdomen.* Stomach distended, with its external surface natural. Small intestines considerably distended, and of a purplish colour. Large intestines in some places distended, in others much contracted, with their external surface natural. The stomach contained a large quantity of whitish muddy fluid; its mucous coat was lined with a white coagulum, and exhibited a blush of red near the pylorus. The duodenum contained a large quantity of a white turbid serum, and a large lumbricus; its mucous coat was of a white colour, and was lined with a whitish mucus throughout its whole extent. The jejunum and ileum contained a large quantity of serous fluid, mixed with flakes of a white coagulum; their mucous membrane had a light vermilion colour, and was lined with a white coagulum. The large intestines contained a considerable quantity of turbid serous fluid. The mucous membrane of the cœcum, and greater part of the colon, had a reddish colour, and was lined with a diaphanous mucus. The lower part of the colon and rectum were healthy. The liver was healthy in its structure, with more blood than usual in its veins. The gall-bladder contained healthy and somewhat inspissated bile. The urinary bladder was healthy.

*Thorax.* Heart natural; blood dark-coloured. Structure of the lungs healthy. A quantity of white froth was found in the trachea. The bronchiæ were filled with a white froth, and a large quantity of grey serous fluid mixed with white flakes.

*Encephalon.* Considerable congestion in all the meningeal veins. All the contents of the cranium were in other respects healthy.

I will leave this case without any remarks upon it at present, as it has been related here only with the view of shewing that the catarrhal affection in cholera sometimes extends to the pulmonary membrane, as well as to the mucous membrane of the other viscera.

I have been able to gain no information on this subject from any dissections that have been already made public, except in one instance. Mr. R. H. England, assistant surgeon, in a report to the Madras Medical Board, in 1821, mentions that, "on cutting into the lungs, a grey fluid oozed from the divided places." This grey fluid could, of course, be nothing but a secretion from the mucous membrane of the air-passages and cells; and, as the remark is a general one, and not confined to any particular case, we must conclude that it was an invariable, or, at least, a very common appearance.

These instances, then, are sufficient to prove that the catarrhal affection frequently extends to the pulmonary mucous membranes, as well as to the other mucous membranes of the body.

The skin also frequently participates in the diseased action of the mucous membranes; for, in many cases, we find it covered with cold clammy sweat, or with a profuse perspiration. If, with Bichat, we view the mucous membranes and the skin merely as different portions of one continuous surface, the functions of which are analogous, we may consider the vitiated perspiration in cholera merely as the effect of the same morbid action, which in different cases extends to every part of this extensive membranous tegument, external as well as internal.

From a careful examination of the cholera secretion, procured from the stomach and intestines of several individuals who died of the disease, I found that it has the following chemical characters and composition: It does not affect litmus or turmeric papers. It becomes of a dark-grey colour when mixed with calomel. It consists of two substances; the one a transparent serous fluid, the other an opaque white coagulum. The former is perfectly soluble in cold water, which enables us easily to separate it from the latter, which is quite insoluble. This separation (which indeed often takes place spontaneously, the coagulum being frequently found diffused in the form of flakes in the serous fluid,) may be considered the first step towards the analysis of the secretion; in the same way that the coagulation and separation of the crassamentum form the first step towards ascertaining the nature of blood.

The following experiments were made on the two substances taken separately.*

I. Serous fluid.

*a.* Tincture of galls produced a precipitate, when added to a mixture of the serous fluid with cold water.

*b.* Alcohol produced a precipitate, when added to the same mixture.

*c.* Muriate of mercury produced a white precipitate.

*d.* Sulphuric acid produced a white precipitate.

*e.* It was coagulated by heat.

*f.* It did not affect litmus paper.

* These experiments were performed two or three times with the same results.

II. Coagulated matter.

*a.* Insoluble in cold water.
*b.* Slightly soluble in boiling water.
*c.* Dissolved when boiled in acetic acid.
*d.* Dissolved by pure aqua ammoniæ.
*e.* Not changed when triturated with calomel.
*f.* Prussiate of potassa, when added to the solution *c*, produced a copious yellow precipitate.

The first set of these experiments proves that the fluid part of the secretion is pure serum, which is particularly confirmed by *d* and *e*. The second set proves that the coagulated part of the secretion is fibrin; test *f* being that which, according to Berzelius, particularly distinguishes that substance. The secretion, therefore, has a composition similar to that of blood deprived of its colouring matter; but the proportions of the serum and fibrin in the secretion are, I imagine, seldom the same as those we find in blood; for, in most cases of cholera, there is an enormous quantity of the serum thrown out by the stomach and intestines, with only a small quantity of coagulated matter.

We must conclude, from these experiments, that the cholera secretion is not merely an increased natural secretion of the mucous membranes, but that while this is increased it is also vitiated; and that it does not originate in an inverted action of the lacteals, as some have conjectured; for, independent of its being very abundant in the stomach and large intestines, where there are few or no lacteals, it has very little resemblance to chyle. The circumstance of its not affecting vegetable colours proves that there is no free acid in the secretion, and thereby shews that Dr. Ainslie's views of the disease cannot be maintained.

The turbid appearance which the serous fluid sometimes has, and the different colours which the secretion occasionally exhibits, ought not to be considered as constituting separate varieties; for, in all probability, they are owing entirely to the admixture of calomel, and other medicines given for the cure of the disease. The creamy or purulent-like matter mentioned above probably differs little from the more common cholera secretion, except in the proportion of its constituent parts. It has a perfectly homogeneous appearance. From a great portion of it not being soluble in cold water, and being precipitated from its solution in acetic acid by prussiate of potassa, it may be inferred that, like the more common secretion, it contains fibrin.

That the disordered state of the mucous membrane is not a partial occurrence, but is invariably present in cholera, is a fact that rests on the very best evidence, viz. on that of Mr. Scot, who mentions it at page 34 of his Report on the Epidemic. "We have seen," says he, "that serous membranes are not necessarily affected in cholera, but that mucous membranes, including the skin, which is of an analogous nature, are affected; and that this affection is, in some part or other, invariable." And again, he says, "The affections of the skin and mucous membranes of the body in cholera are evidenced by a cold relaxed condition of the former, and, in the latter, by the state of the stomach and intestines, from all of which a watery or mucous discharge is largely poured out; and by the state of the bladder and ureters, which are found to be coated with a mucus similar to that which is observed in the other passages. The fluid discharged at times from the bladder is almost always stated to be limpid, colourless, and in small quantity; leading to the inference that it may not be urine, but a mere watery exudation from the lining membrane. That the mucous membranes are affected is further evidenced by the moist state of the mouth, even under the most urgent thirst; and by the state of the eyes, where there appears to be a peculiar secretion, or exudation, in the form of a film."

Many cases have been recorded, in which we find it stated that, on examination, no morbid appearances could be detected. But, in these cases, were the mucous membranes carefully examined? From what has been stated in a former page, we have every reason to suspect they were not. Even if they were carefully examined, and no morbid appearances could be discovered, we are not the less certain that their *functions* were disordered during life; for of this the copious morbid secretions thrown off by vomiting and stool afford a sufficient proof. It is stated, in case 8th, in the Bengal Report on the Epidemic Cholera, that no morbid appearances could be discovered on dissection: yet, even supposing this dissection to have been made with the greatest care, it is perfectly evident that there was great functional derangement of the gastro-enteric mucous membrane during life; for the most prominent symptoms were frequent vomiting and purging.

The catarrhal affection has its seat generally, I think, in the stomach and small intestines; and in almost all severe and protracted cases, it appears to pervade every mucous membrane of the body. The stomach is certainly most obnoxious to the disease, and I have met with no case in which it was free from it. I imagine that the mucous membrane of the air-passages is not always affected; but it frequently is so, and perhaps invariably, in severe cases.

It has been already mentioned, in describing the symptoms of the disease, that cases have occasionally occurred in which catarrhal cholera has been followed by great excitement and by various symptoms of gastro-enteric inflammation. The only full account of these cases has been given by Mr. Jameson, in his Report drawn up under the superintendence of the Medical Board of Bengal, and I therefore extract the following valuable observations on the autopsy of the disease from that work: "The bodies of such as had sunk in the earlier stages of the malady frequently exhibited hardly any unhealthy appearance. This was more especially observable among Europeans of weak and sickly constitutions, and among natives of the poorer classes. On laying open the bodies of such persons, it was remarked that the abdomen emitted a peculiar offensive odour, very different from the ordinary smell of dead subjects. In them there was not the slightest mark of previous increased vascular action throughout the whole intestinal canal; which rather appeared paler than usual and flabby, and was filled with an amazing quantity of whitish or muddy fluid, or empty and inflated with air. Sometimes in the stomach this fluid was found mixed with pieces of curdled matter, or lumps of undigested food. This appearance of relaxation was not confined to those in whom the spasmodic affections were absent. It occurred frequently where the cramps of the abdominal muscles had been violent, and the pain in the stomach excruciatingly severe.

"On laying open the abdomen of such as had lived some time after the commencement of the attack, and especially of Europeans and the stouter natives, a different set of appearances was brought into view. The omentum and mass of intestines were often found displaced, and preternaturally vascular, with partial adherence between the diaphragm, liver, and surrounding viscera. The colour of the intestines varied from deep rose to a dark hue, according as the increased vascular action had been arterial or venous. In some instances the outer surface of the stomach, likewise, was florid, and its veins tinged with black blood; but this was not so in the generality of cases.

This organ was, however, much contracted, and its substance hard, and frequently thickened. On cutting into it, it was found sometimes empty, sometimes partially, and at others largely, filled with fluid of various colour and consistence, thin and transparent, milky, green, dark, grumous or muddy.

Sometimes this fluid was black, like lamp-black; sometimes it consisted of pure blood; and at other times, of blood mixed with bile. On removing this, the inner surface was frequently seen lined with coagulated lymph, bloody gelatine, or a muddy glossy viscid matter; which, on being washed away, brought the highly inflamed coats into view. Of these the appearance was various; generally they were crossed by streaks of a deep red, interspersed with spots of inflammation made up of tissues of enlarged vessels. Sometimes the inflammation was florid and bright-coloured, so as to give the whole inner surface of the organ the appearance of a minutely injected anatomical preparation. In some instances, ulceration had begun, and the villous coat was partially abraded; in others, incipient mortification had occurred, and the coats were puckered into net-work, or drawn into folds, with patches of red near the pylorus, &c.

In cases of several days' standing, the inner coat of the small guts was ulcerated, and they were filled with sanies, having portions of lymph floating in it. Then the large intestines were lined with a dark, thick, pitchy stuff poured out from the liver, as it had begun to renew its action."*

